# Supplementary material for: Development of ferret immune repertoire reference resources and single-cell-based high-throughput profiling assays
Source: J Virol. 2025 Mar 21;99(4):e00181-25. doi: 10.1128/jvi.00181-25 (PMC11998538; doi:10.1128/jvi.00181-25)
Supplement: Supplemental material — Figures S1 to S6, Table S5, supplemental text, and legends for additional supplemental tables. [file jvi.00181-25-s0001.pdf]

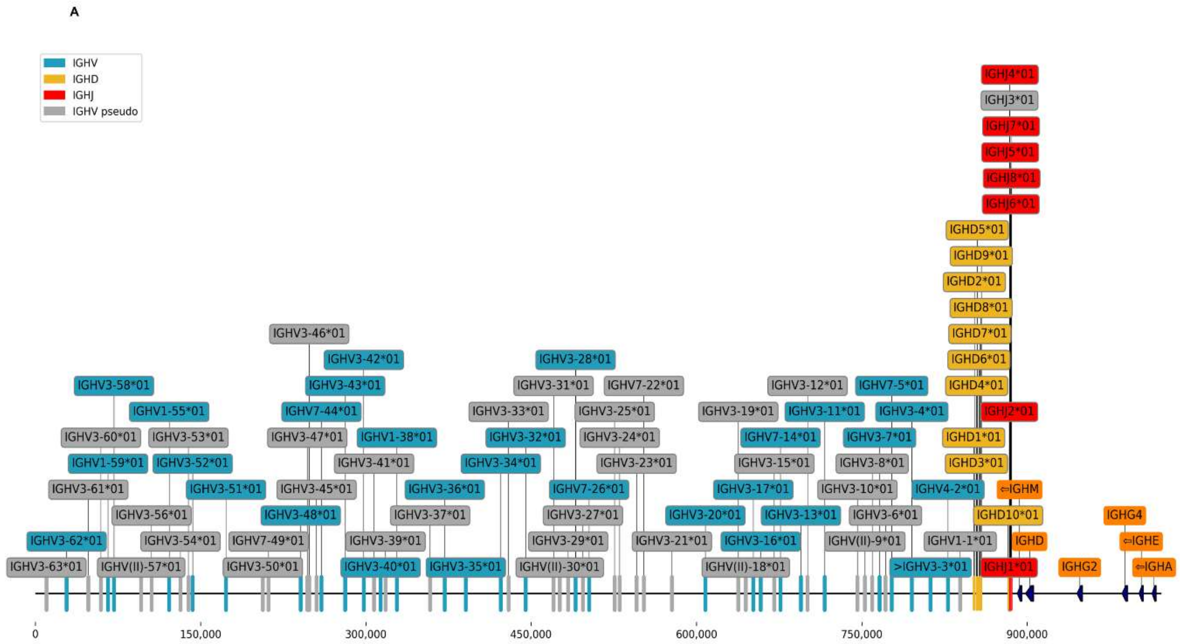

Figure S1. Genomic organization of ferret Ig regions. (A) Heavy chains and associated V, D, and J genes.

B

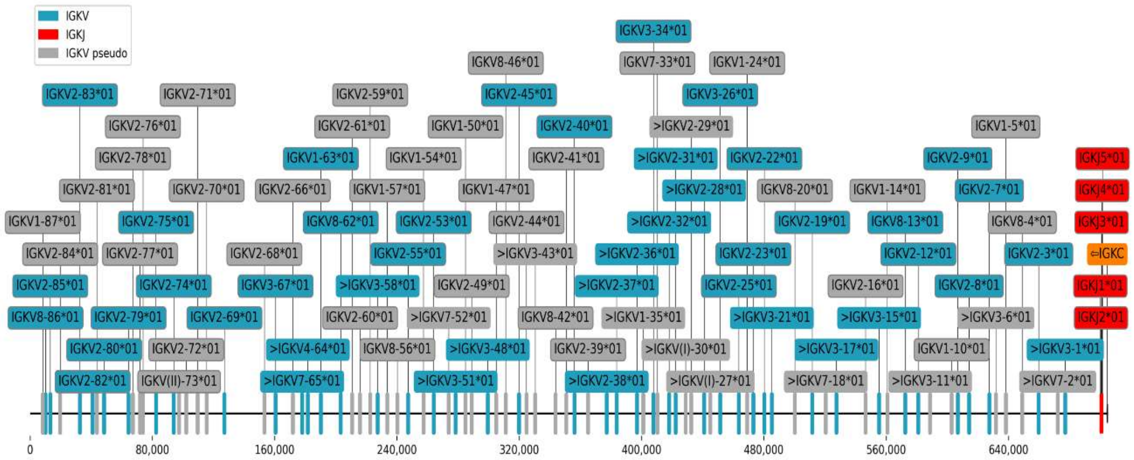

Figure S1. Genomic organization of ferret Ig regions. (B) Kappa light chains and associated V and J genes.

C

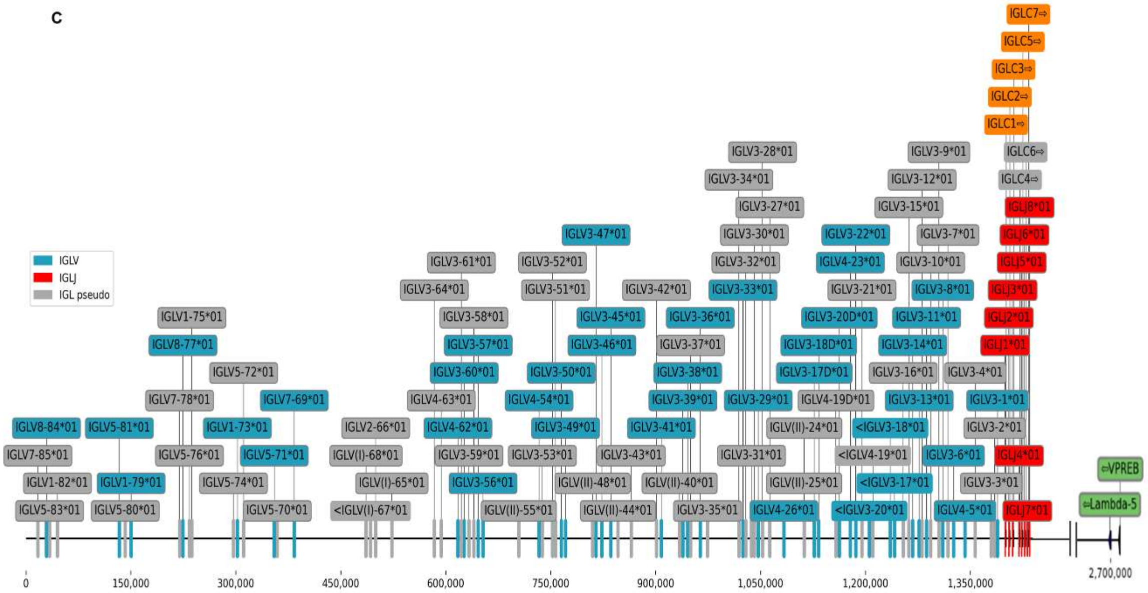

Figure S1. Genomic organization of ferret Ig regions. (C) Lambda light chains and associated V and J genes.

D

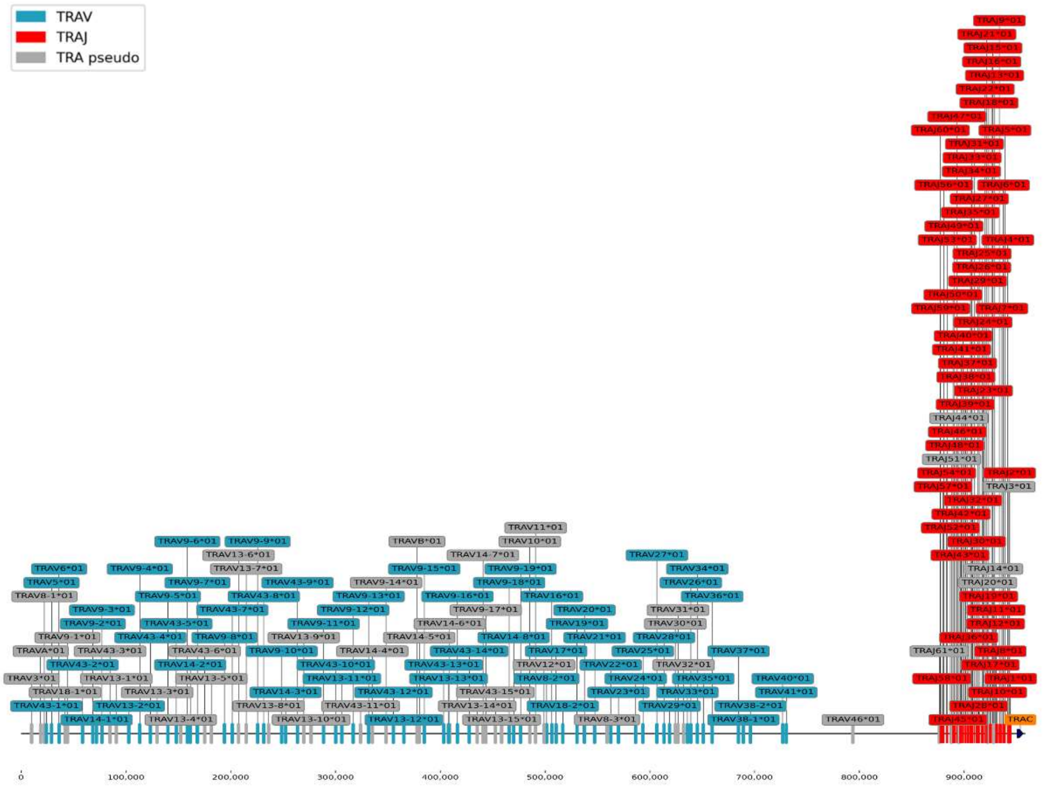

Figure S1. Genomic organization of ferret TCR region. (D) TRA associated V, D and J genes.

E

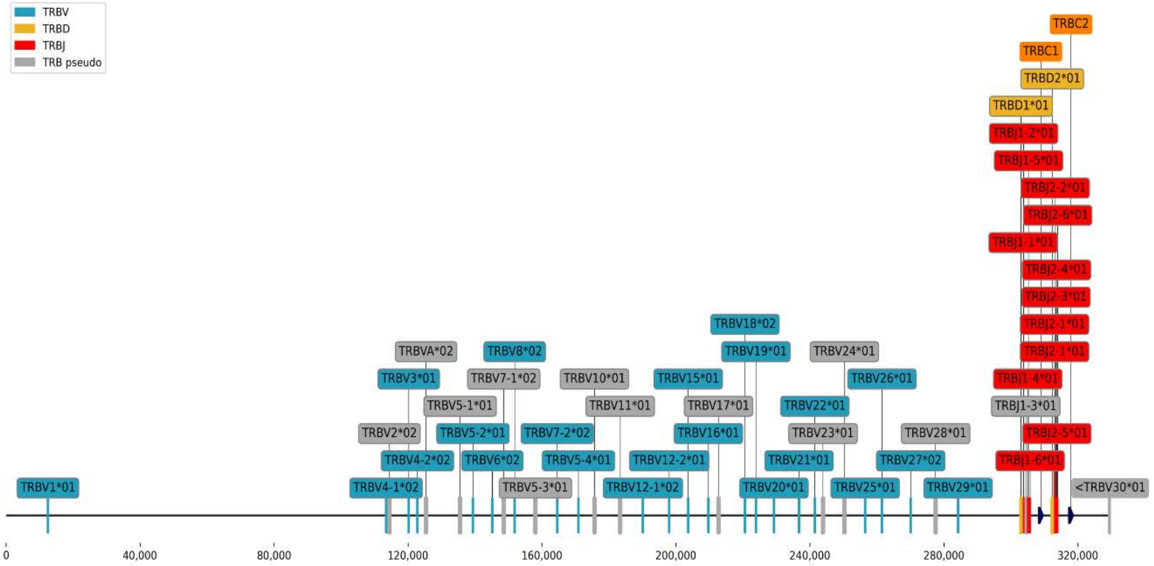
TRBV
TRBD
TRBJ
TRB pseudo
TRBC2
TRBC1
TRBD2*01
TRBD1*01
TRBJ1-2*01
TRBJ1-5*01
TRBJ2-2*01
TRBJ2-6*01
TRBJ1-1*01
TRBJ2-4*01
TRBJ2-3*01
TRBJ2-1*01
TRBJ2-1*01
TRBJ1-4*01
TRBJ1-3*01
TRBJ2-5*01
TRBJ1-6*01
<TRBV30*01
TRBV18*02
TRBVA*02
TRBV8*02
TRBV19*01
TRBV24*01
TRBV3*01
TRBV7-1*02
TRBV10*01
TRBV15*01
TRBV26*01
TRBV5-1*01
TRBV11*01
TRBV17*01
TRBV22*01
TRBV2*02
TRBV5-2*01
TRBV7-2*02
TRBV16*01
TRBV23*01
TRBV28*01
TRBV4-2*02
TRBV6*02
TRBV5-4*01
TRBV12-2*01
TRBV21*01
TRBV27*02
TRBV1*01
TRBV4-1*02
TRBV5-3*01
TRBV12-1*02
TRBV20*01
TRBV25*01
TRBV29*01
0
40,000
80,000
120,000
160,000
200,000
240,000
280,000
320,000

F

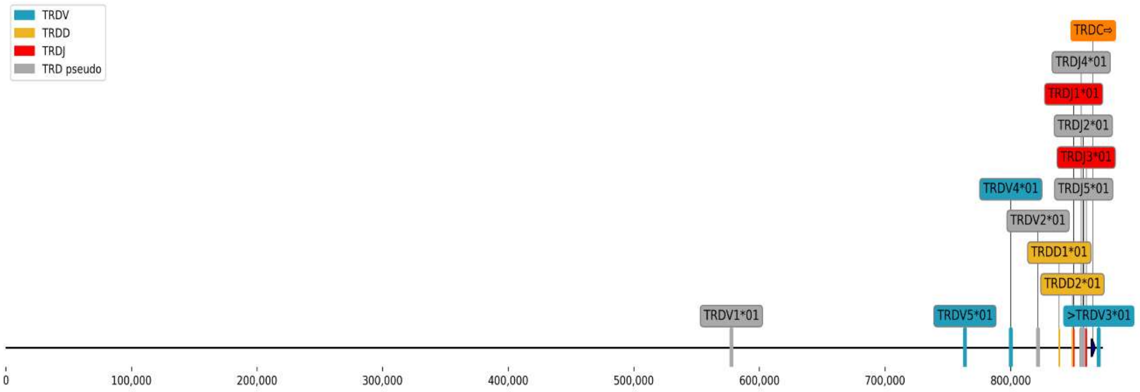
TRDV
TRDD
TRDJ
TRD pseudo
TRDC⇨
TRDJ4*01
TRDJ1*01
TRDJ2*01
TRDJ3*01
TRDV4*01
TRDJ5*01
TRDV2*01
TRDD1*01
TRDD2*01
TRDV1*01
TRDV5*01
>TRDV3*01
0
100,000
200,000
300,000
400,000
500,000
600,000
700,000
800,000

G

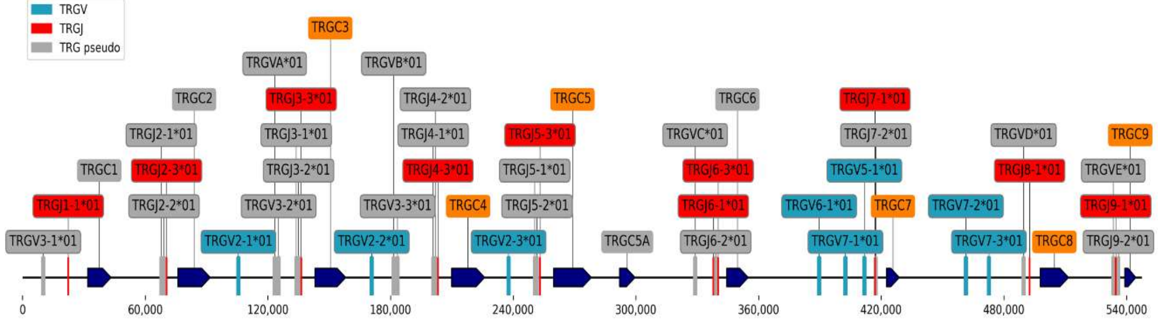
TRGV
TRGJ
TRG pseudo
TRGC3
TRGVA*01
TRGVB*01
TRGC2
TRGJ3-3*01
TRGJ4-2*01
TRGC5
TRGC6
TRGJ7-1*01
TRGJ2-1*01
TRGJ3-1*01
TRGJ4-1*01
TRGJ5-3*01
TRGVC*01
TRGJ7-2*01
TRGVD*01
TRGC9
TRGC1
TRGJ2-3*01
TRGJ3-2*01
TRGJ4-3*01
TRGJ5-1*01
TRGJ6-3*01
TRGV5-1*01
TRGJ8-1*01
TRGVE*01
TRGJ1-1*01
TRGJ2-2*01
TRGV3-2*01
TRGV3-3*01
TRGC4
TRGJ5-2*01
TRGJ6-1*01
TRGV6-1*01
TRGC7
TRGV7-2*01
TRGJ9-1*01
TRGV3-1*01
TRGV2-1*01
TRGV2-2*01
TRGV2-3*01
TRGC5A
TRGJ6-2*01
TRGV7-1*01
TRGV7-3*01
TRGC8
TRGJ9-2*01
0
60,000
120,000
180,000
240,000
300,000
360,000
420,000
480,000
540,000

Figure S1. Genomic organization of ferret TCR region. (E) TRB and associated V, D, and J genes; (F) TRD and associated V and J genes; (G) TRG and associated V and J genes.

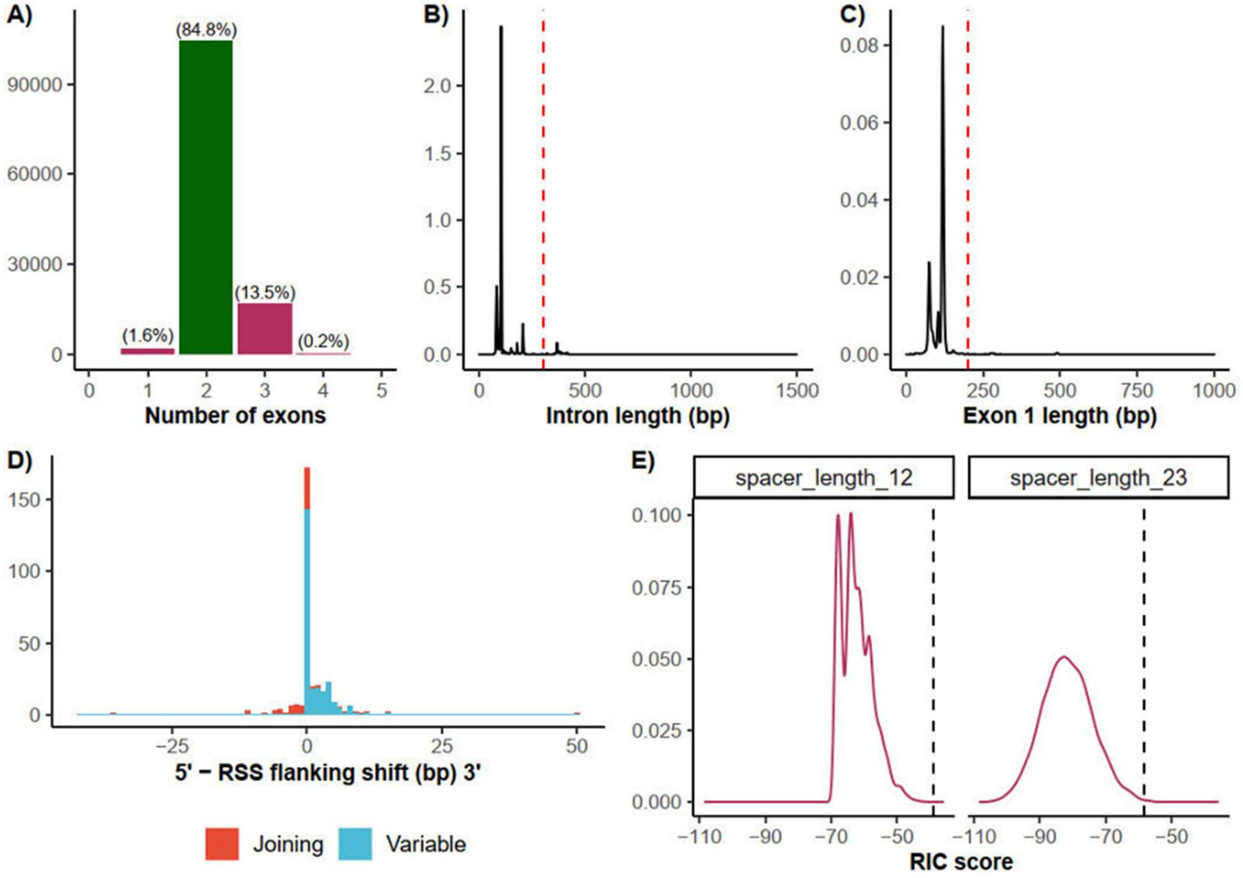

Figure S2. Identification, filtering, and annotation of putative V- and J-region genes using full-length CCS reads. A) Distribution of the number of exons from aligned V-region sequences to the ferret reference assembly. Only sequence alignments with two exons were kept for downstream analyses. B) Distribution of intron length between the two aligned V-region exons. The red line at 350 base pairs indicates our filtering threshold. C) Distribution of the length of the first exon of V-region sequence alignments. The red line indicates at 200 base pairs indicates our filtering threshold. D) Distribution of the number of bases that were shifted to the end or start positions of putative V- and J-region genes, based on the flanking recombination signal sequences (RSSs). We observed a median shift of V-region sequences of 0 base pairs and a median of -1 for J-region sequences In some instances, J-region sequences were trimmed back because the RSS was contained within the originally aligned J-region sequences (i.e negative shift values). E) The RSS score distributions of randomly selected sequences in the ferret genome that started with a CA and the same RSS score thresholds were set as in previous studies (1, 2).

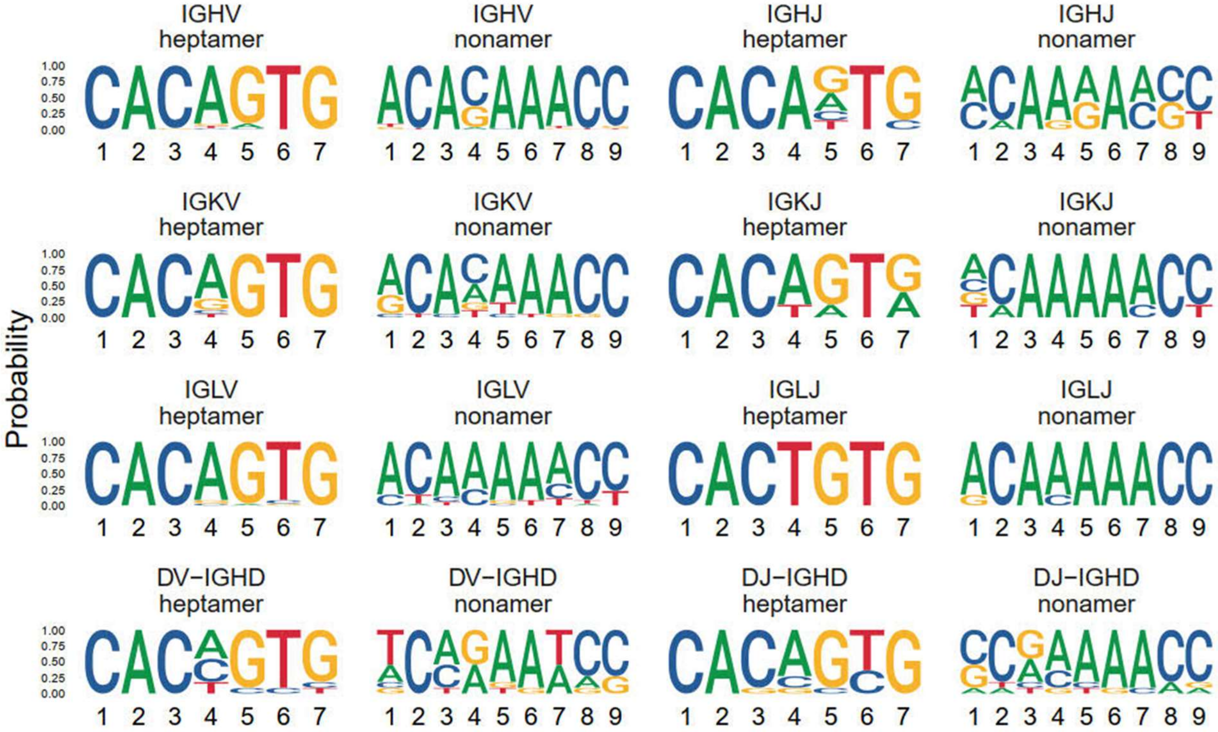

Figure S3. (A) Recombination signal sequences of V, D and J genes (V-RS, 5'D-RS, 3'D-RS and J-RS respectively) for each IG locus of the domestic ferret. The height of symbols indicates the relative frequency of each nucleotide at that position.

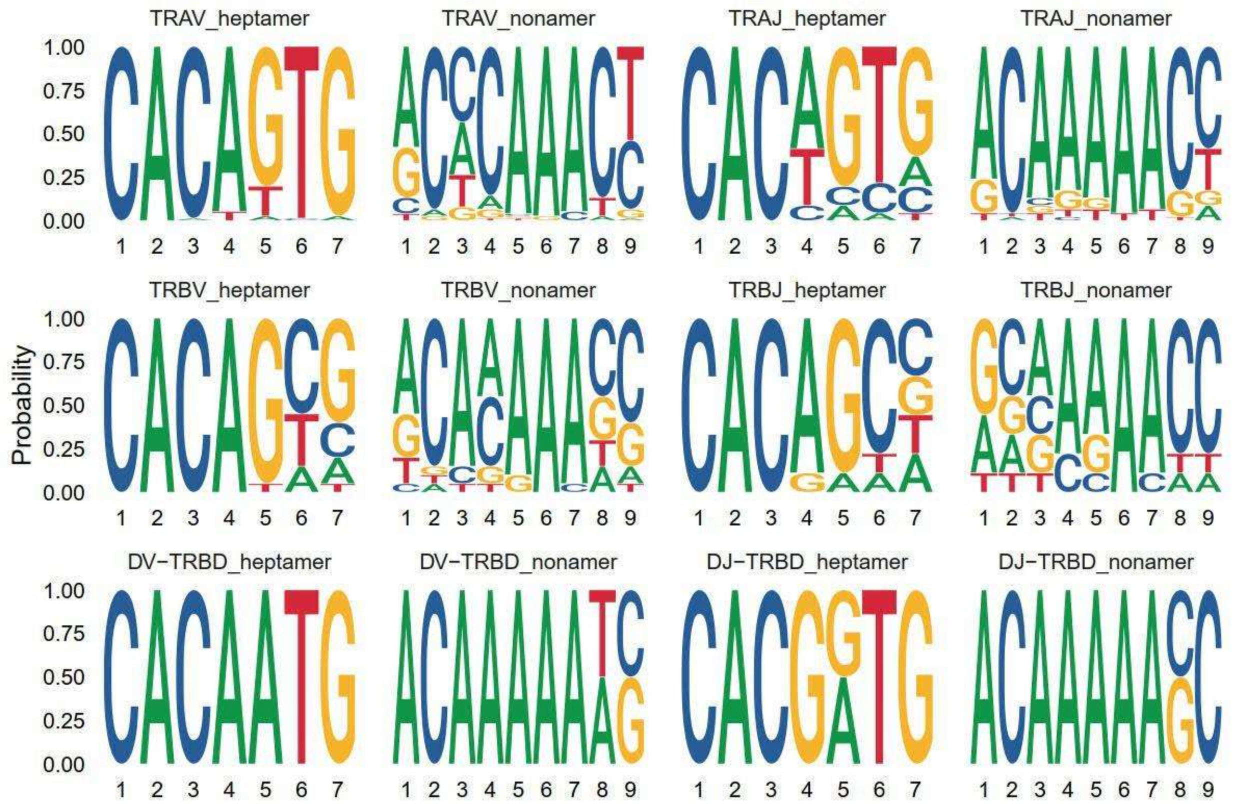

Figure S3. (B) Recombination signal sequences of V, D and J genes (V-RS, 5’D-RS, 3’D-RS and J-RS respectively) for the TCRA and TCRB loci of the domestic ferret. The height of symbols indicates the relative frequency of each nucleotide at that position.

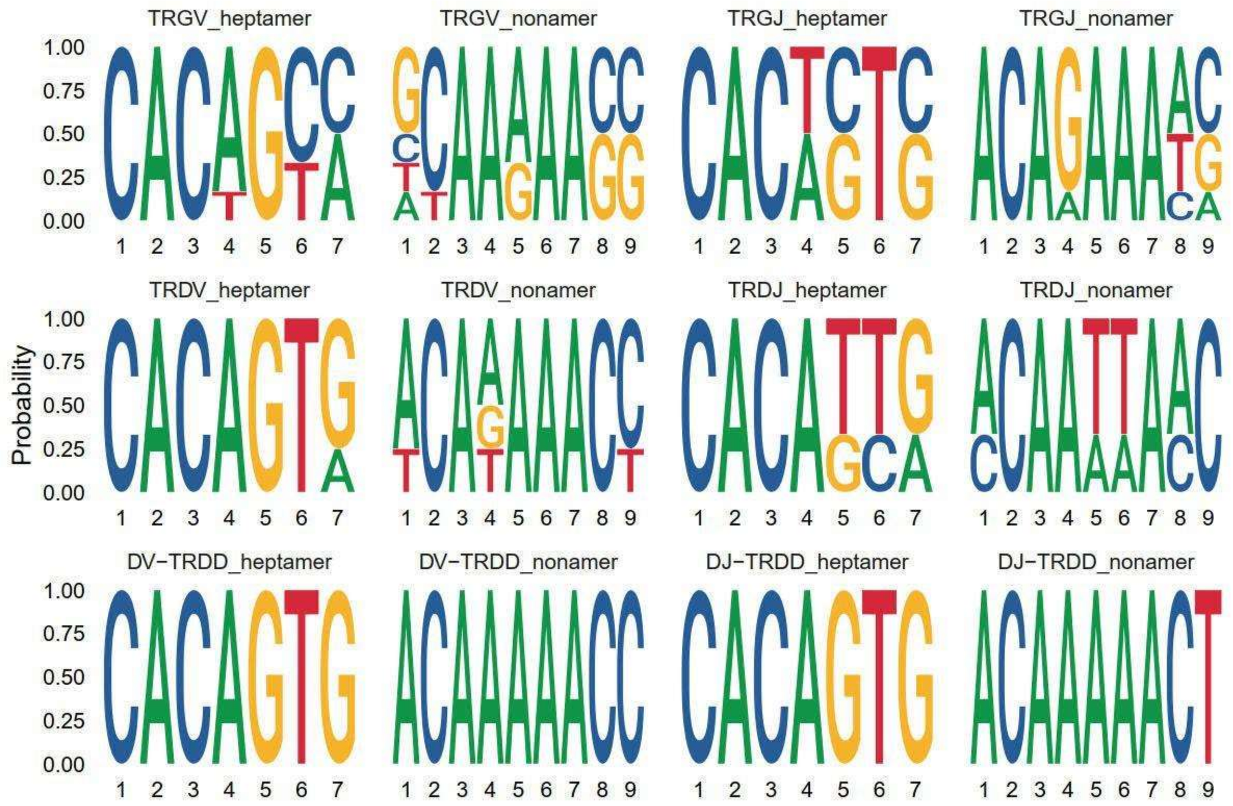

Figure S3. (C) Recombination signal sequences of V, D and J genes (V-RS, 5'D-RS, 3'D-RS and J-RS respectively) for the TCRG and TCRD loci of the domestic ferret. The height of symbols indicates the relative frequency of each nucleotide at that position.

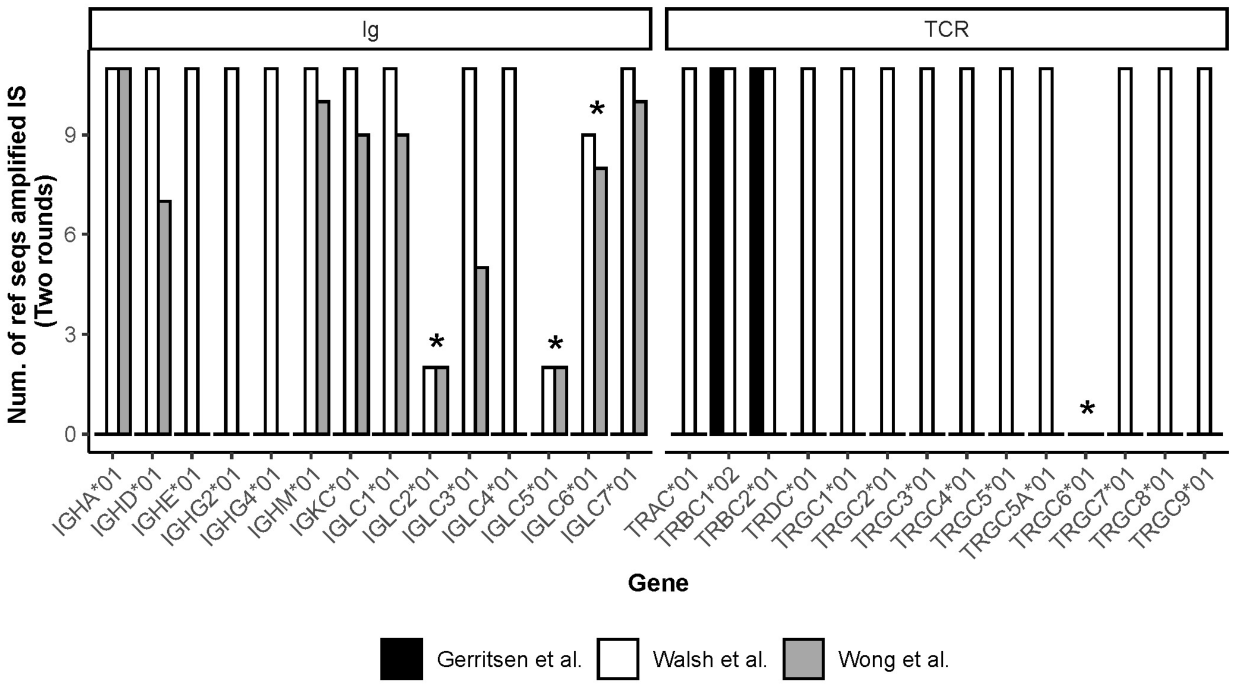

Figure S4. In silico PCR analysis of ferret-specific Ig and TCR primers. Primers from Wong et al. (Wong et al., 2020) and Gerritsen et al (3) and primers described in this manuscript, were assessed in silico. Both inner and outer primers were tested from each set. Shown are the number of ferret C-region reference sequences amplified in silico. For a sequence to be considered amplified, we required that it was amplified by both inner and outer reverse primers. Left: Amplification of Ig reference sequences using primers from Wong et al (4) and primers described in this manuscript. Right: Amplification of TCR reference sequences using primers from Gerritsen et al (3) and primers described in this manuscript. For the isPcr tool we used the parameters which required a 14-nucleotide perfect match from the 3' end of the primer (-tileSize=11 -minGood=14 -minPerfect=14).

*: IGLC2*01 and IGLC5*01 were identified on 2 ferret genome assemblies in total. IGLC6*0.1 was identified on 9 ferret genome assemblies in total. TRGC6*01 is a pseudogene with a truncated exon 1 sequence that does not have the inner primer binding site, therefore no amplification. Additional details are available on the GitHub: https://github.com/ncsu-penglab/FerretIgTCR/tree/main/Annotations.

A

PBMC

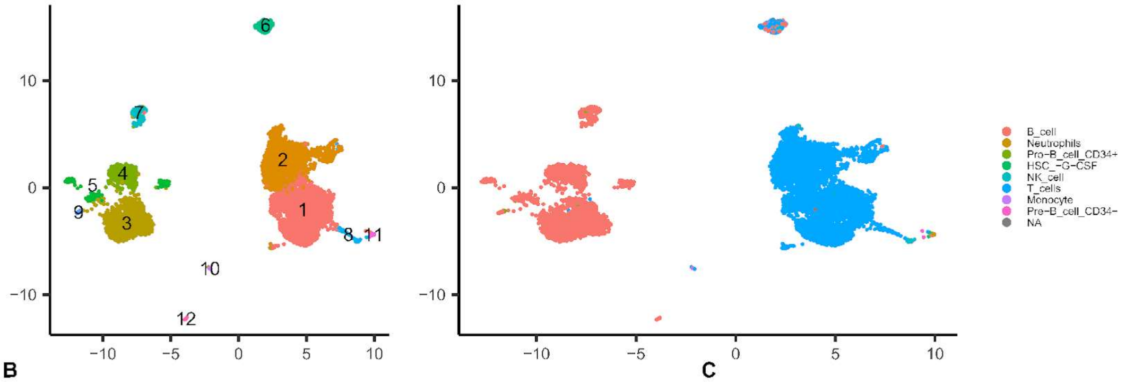

B

SingleR	1	2	3	4	5	6	7	8	9	10	11	12
B_cell	2	2	1451	572	309	73	224	0	41	0	0	24
Neutrophils	0	0	0	0	0	0	0	0	0	0	17	0
Pro−B_cell_CD34+	0	0	10	6	2	0	5	0	1	0	0	0
HSC_−G−CSF	4	0	0	0	0	0	0	0	0	0	4	0
NK_cell	0	8	0	0	0	3	0	32	0	0	0	0
T_cells	2770	2125	10	0	1	211	1	49	0	1	0	0
Monocyte	0	0	1	0	0	0	0	0	0	14	2	0
Pre−B_cell_CD34−	2	3	3	0	0	2	1	2	0	10	1	0

C

SingleR	None	VDJ	VDJ,VJ	VJ
B_cell	0	0	0	0
Neutrophils	0	0	0	0
Pro−B_cell_CD34+	0	0	0	0
HSC_−G−CSF	0	0	0	0
NK_cell	29	0	3	0
T_cells	4	7	31	7
Monocyte	0	0	0	0
Pre−B_cell_CD34−	2	0	0	0

D

Splenocyte

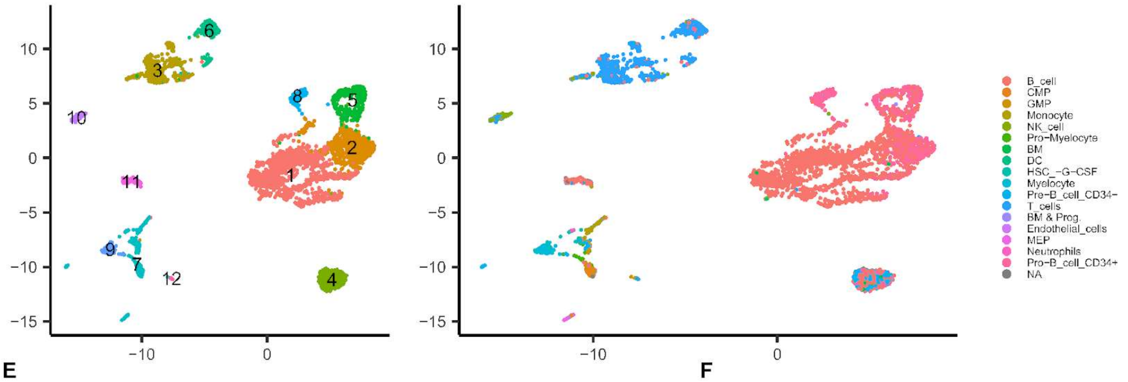

E

SingleR	1	2	3	4	5	6	7	8	9	10	11	12
B_cell	1306	392	20	169	148	14	7	3	0	2	70	5
CMP	0	0	0	0	0	0	38	0	0	0	0	0
GMP	1	0	0	0	0	0	43	0	0	0	0	1
Monocyte	4	0	0	0	0	0	46	0	0	0	2	1
NK_cell	0	0	34	0	0	18	0	1	0	50	0	0
Pro−Myelocyte	0	0	0	0	0	0	19	0	1	0	0	0
BM	1	1	0	25	0	0	1	0	0	0	0	0
DC	2	0	0	0	0	0	0	0	0	0	0	0
HSC_−G−CSF	0	0	0	0	0	1	9	0	9	0	0	0
Myelocyte	0	0	0	0	0	0	11	0	87	0	0	0
Pre−B_cell_CD34−	0	0	2	143	0	1	31	0	6	2	0	12
T_cells	1	0	444	16	1	227	3	1	0	44	8	0
BM & Prog.	0	0	0	1	0	0	4	0	0	0	0	0
Endothelial_cells	0	0	0	0	1	0	0	0	0	0	0	0
MEP	0	0	0	0	0	0	27	0	0	0	0	0
Neutrophils	0	0	0	0	1	0	8	0	0	0	0	0
Pro−B_cell_CD34+	65	281	2	83	202	3	4	119	0	1	4	0

F

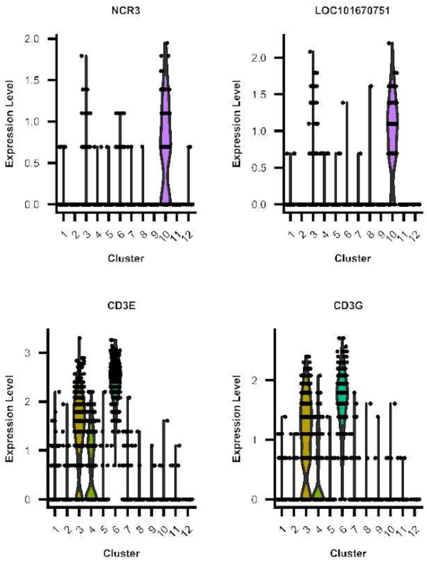

Figure S5. Summary of the cell type prediction for each cell using SingleR (Aran et al., 2019). (A) UMAP plots of the assignment of cell cluster (left) and cell type prediction (right) in the ferret PBMC sample. (B) Number of predicted cell types in each cluster of the ferret PBMC sample. (C) TCR VDJ-VJ pairing for each predicted cell type in the cluster 8 of the ferret PBMC sample. (D) UMAP plots of the assignment of cell cluster (left) and cell type prediction (right) in the ferret splenocyte sample. (E) Number of predicted cell types in each cluster of the ferret splenocyte sample. (F) Expressions of selected cell marker genes in the individual cell clusters of the splenocyte sample. NCR3 and NCR1 (LOC101670751) are NK cell markers. CD3E and CD3G are T cell markers.

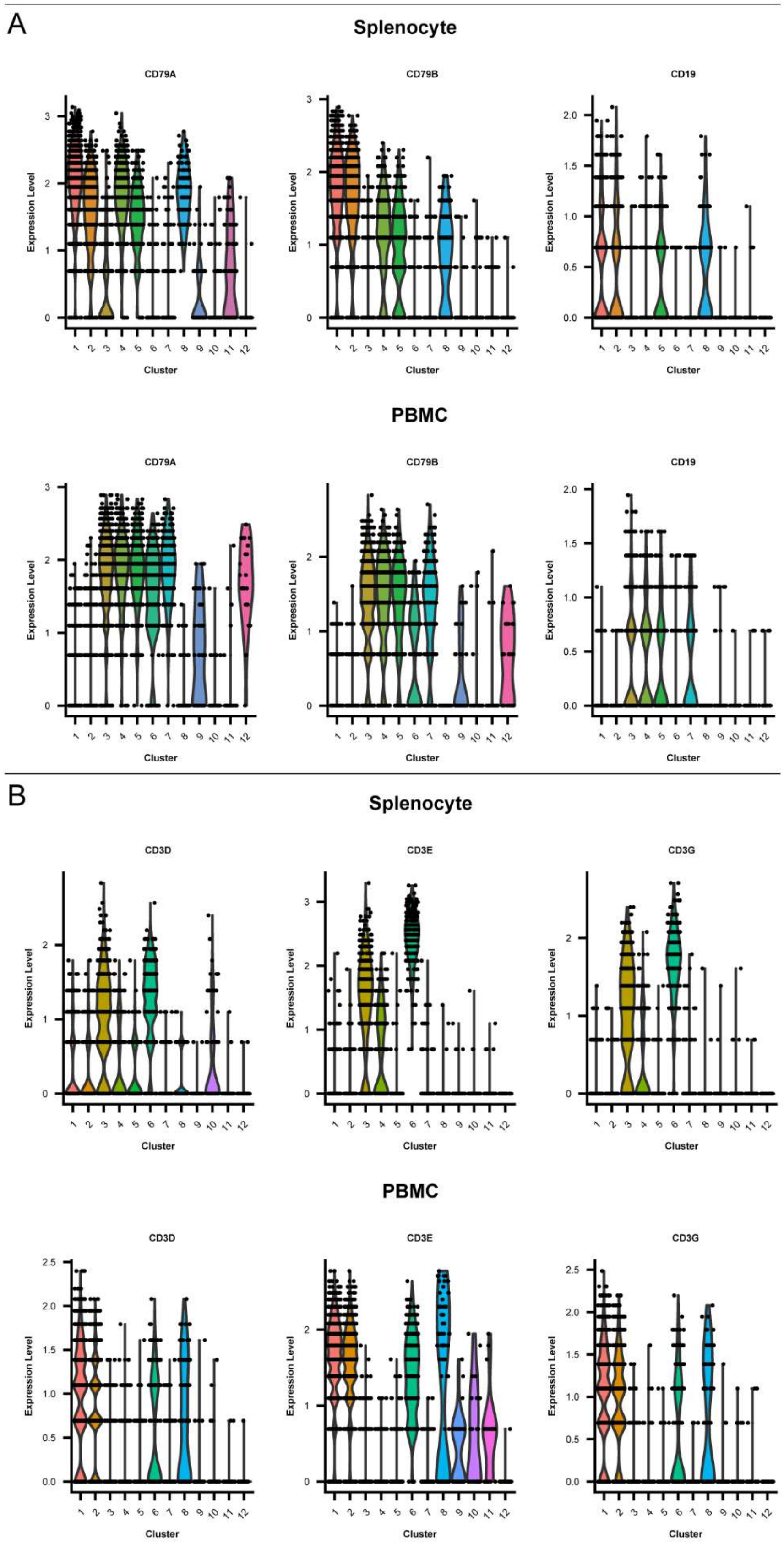

Figure S6. Expression of canonical B (**A**) and T (**B**) cell markers in the individual cell clusters of ferret PBMC and splenocyte samples.

Table S5. Comparisons of ferret cell type predictions using human references.

The ferret cell types were predicted using three cell type reference datasets from the celldex package which included two general-purpose human references: Human primary cell atlas (HPCA) and Blueprint/ENCODE, and one immune reference Immunological Genome Project (ImmGen). The table below shows the number of predicted cell types in each cluster of the ferret PBMC and splenocyte samples, separately for each prediction. The numbers highlighted in green show that there was an overall agreement between the predictions of main cell types such as B and T cells using two general-purpose human references. The numbers highlighted in red indicate potential disagreements among cell type predictions. One notable difference was cluster # 8 of the splenocyte sample. The prediction using Blueprint/ENCODE suggested cluster # 8 was a mixture of HSC and B cells, but our Ig analysis and B cell marker expression indicated they were pro-B cells and agreed with the predictions using Human primary cell atlas (HPCA). Another notable difference was cluster # 4 of the splenocyte sample. The prediction using Blueprint/ENCODE suggested cluster # 4 was a mixture of B and T cells, while the prediction using Human primary cell atlas (HPCA) suggested cluster # 4 was B cells. Cluster # 4 appeared to have high expression of B cell markers CD79A and CD79B and low expression of T cell markers (Figure S5). The prediction using ImmGen appeared to be incorrect overall as all cell clusters were classified as DCs.

To confirm cluster #4 was likely a B cell cluster, we also approximated the detection of Ig transcripts using the RNA-seq reads aligned to the ferret genomics regions annotated with Ig variable genes and calculated the percentage of cells in each cluster with Ig transcripts detected (see the table and barplots after the cell type prediction table).

Sample: PBMC, Reference: Human primary cell atlas (HPCA)												
	**1**	**2**	**3**	**4**	**5**	**6**	**7**	**8**	**9**	**10**	**11**	**12**
B_cell	2	2	1451	572	309	73	224	0	41	0	0	24
HSC_-G-CSF	4	0	0	0	0	0	0	0	0	0	4	0
Monocyte	0	0	1	0	0	0	0	0	0	14	2	0
Neutrophils	0	0	0	0	0	0	0	0	0	0	17	0
NK_cell	0	8	0	0	0	3	0	32	0	0	0	0
Pre-B_cell_CD34-	2	3	3	0	0	2	1	2	0	10	1	0
Pro-B_cell_CD34+	0	0	10	6	2	0	5	0	1	0	0	0
T_cells	2770	2125	10	0	1	211	1	49	0	1	0	0
NA	5	3	4	0	1	4	0	0	0	0	0	0
Sample: PBMC, Reference: Blueprint/ENCODE												
	**1**	**2**	**3**	**4**	**5**	**6**	**7**	**8**	**9**	**10**	**11**	**12**
B-cells	0	1	1466	576	312	30	229	0	39	0	0	24
CD4+ T-cells	1293	1237	2	0	0	59	1	2	0	0	0	0
CD8+ T-cells	1482	895	8	0	1	203	1	51	3	6	4	0
Monocytes	0	0	3	1	0	0	0	0	0	19	6	0
Neutrophils	0	0	0	0	0	0	0	0	0	0	14	0
NK cells	0	5	0	0	0	1	0	30	0	0	0	0
NA	8	3	0	1	0	0	0	0	0	0	0	0
Sample: PBMC, Reference: Immunological Genome Project (ImmGen)												
	**1**	**2**	**3**	**4**	**5**	**6**	**7**	**8**	**9**	**10**	**11**	**12**
B cells	3	4	3	1	0	0	0	0	0	1	0	1
B cells, pro	61	86	4	0	1	1	1	2	1	0	0	0
DC	2705	2036	1450	570	309	287	223	79	41	10	21	22
Endothelial cells	3	9	16	4	1	0	4	1	0	0	0	0
Eosinophils	0	2	2	2	0	2	0	0	0	12	2	0

Epithelial cells	0	1	0	0	0	0	0	0	0	0	0	0
ILC	3	1	1	0	1	0	1	0	0	0	0	0
Microglia	7	2	3	1	1	3	1	1	0	0	1	1
NA	1	0	0	0	0	0	1	0	0	2	0	0

Sample: Splenocyte, Reference: Human primary cell atlas (HPCA)												
	**1**	**2**	**3**	**4**	**5**	**6**	**7**	**8**	**9**	**10**	**11**	**12**
B_cell	1306	392	20	169	148	14	7	3	0	2	70	5
BM	1	1	0	25	0	0	1	0	0	0	0	0
BM & Prog.	0	0	0	1	0	0	4	0	0	0	0	0
CMP	0	0	0	0	0	0	38	0	0	0	0	0
DC	2	0	0	0	0	0	0	0	0	0	0	0
Endothelial_cells	0	0	0	0	1	0	0	0	0	0	0	0
GMP	1	0	0	0	0	0	43	0	0	0	0	1
HSC_-G-CSF	0	0	0	0	0	1	9	0	9	0	0	0
MEP	0	0	0	0	0	0	27	0	0	0	0	0
Monocyte	4	0	0	0	0	0	46	0	0	0	2	1
Myelocyte	0	0	0	0	0	0	11	0	87	0	0	0
Neutrophils	0	0	0	0	1	0	8	0	0	0	0	0
NK_cell	0	0	34	0	0	18	0	1	0	50	0	0
Pre-B_cell_CD34-	0	0	2	143	0	1	31	0	6	2	0	12
Pro-B_cell_CD34+	65	281	2	83	202	3	4	119	0	1	4	0
Pro-Myelocyte	0	0	0	0	0	0	19	0	1	0	0	0
T_cells	1	0	444	16	1	227	3	1	0	44	8	0
NA	0	0	1	13	0	0	2	0	0	0	8	1
Sample: Splenocyte, Reference: Blueprint/ENCODE												
	**1**	**2**	**3**	**4**	**5**	**6**	**7**	**8**	**9**	**10**	**11**	**12**
Adipocytes	0	0	0	0	1	0	0	0	0	0	0	0
B-cells	1368	666	7	295	257	4	12	63	1	1	77	10
CD4+ T-cells	2	0	156	2	0	3	0	0	0	0	1	0
CD8+ T-cells	2	1	319	107	4	213	10	4	3	23	6	0
DC	2	0	0	0	0	0	1	0	0	0	0	0
Eosinophils	0	0	0	0	0	0	0	0	4	0	0	0
Erythrocytes	0	0	0	1	7	0	23	0	0	0	0	0
HSC	1	6	2	28	83	1	115	57	4	0	1	1
Macrophages	0	0	0	0	0	0	1	0	0	0	0	0
Monocytes	3	1	0	10	1	0	82	0	32	2	2	9
Neutrophils	0	0	0	0	0	0	9	0	58	0	0	0
NK cells	0	0	18	0	0	43	0	0	0	72	0	0
NA	2	0	1	7	0	0	0	0	1	1	5	0
Sample: Splenocyte, Reference Immunological Genome Project (ImmGen)												

	**1**	**2**	**3**	**4**	**5**	**6**	**7**	**8**	**9**	**10**	**11**	**12**
B cells	2	0	0	1	6	2	4	1	0	1	0	0
Basophils	0	0	0	0	0	0	0	0	2	0	0	0
DC	1369	669	487	440	338	258	175	116	41	98	90	13
Endothelial cells	5	5	1	1	3	2	4	6	0	0	2	1
Eosinophils	1	0	0	4	1	0	56	0	52	0	0	6
Epithelial cells	0	0	0	0	1	0	0	0	0	0	0	0
Mast cells	1	0	1	2	2	0	5	1	0	0	0	0
Microglia	2	0	14	2	2	2	5	0	4	0	0	0
NA	0	0	0	0	0	0	4	0	4	0	0	0

The table below shows the percentage of cells in each splenocyte cell cluster with Ig transcripts detected by RNA-seq, based on the RNA-seq reads aligned to the genomic regions annotated with Ig variable genes (IgH: IGHV, IgL: IGLV and IGKV). See main text and methods for additional information. For visual comparison, the same data in the table is also shown by the barplots below. Filtering with the minimum read counts of 3,4,5 showed similar results.

minimum read count	Ig chain type	Splenocyte cell cluster #											
		1	2	3	**4**	5	6	7	8	9	10	11	12
3	IgH	87.2	93	13.1	**99.8**	97.7	40.9	20.2	75	8.7	13.1	93.5	25
3	IgL	90.1	48.2	14.3	**99.8**	25.5	18.6	26.9	14.5	10.7	19.2	100	20
4	IgH	82	87.8	11.3	**99.8**	97.7	29.2	17	66.9	8.7	12.1	93.5	15
4	IgL	87.8	38.7	12.5	**99.8**	17.3	15.2	22.9	8.9	10.7	15.2	100	10
5	IgH	74.1	81.9	10.3	**99.8**	96.9	22	13	63.7	6.8	9.1	93.5	15
5	IgL	85.4	32.9	11.5	**99.8**	15.3	12.9	20.6	7.3	10.7	12.1	100	10

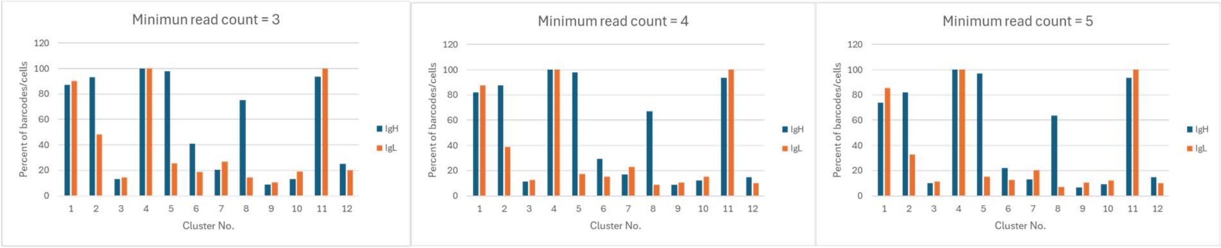

Supplementary Text – cDNA support of ferret gene annotations

Methodology

Concerning the expression of V, D, J genes, Iso-Seq cDNA sequences (YAN12153A1, YAN12802A1, UDEL-6_5, UDEL-8_5 from our BioProject accession # PRJNA939558: https://www.ncbi.nlm.nih.gov/bioproject/939558) were submitted to IMGT/HighV-QUEST (1.9.5 (22 May 2024)) (5) and compared to IMGT ferret reference directory (202430-2 (23 July 2024)). The number of all productive cDNA sequences (https://www.imgt.org/IMGTScientificChart/SequenceDescription/IMGTfunctionality.html) matching the V, D, and J genes and alleles without trimmed nucleotides in 5' and 3' or mismatches (deduced from the CIGAR format provided in the IMGT/HighV-QUEST results) were recorded per gene and are shown in Table S2.

The IMGT reference directory is composed of all ferret alleles of the assembly GCF_011764305.1, the current NCBI reference assembly. Additionally, the reference directory comprises TRB genes of the assembly: GCF_000215625.1 published in Pégorier, Perrine et al. (6) Moreover, concerning IGH, some genes found in GCA_010015535.1 and identified in transcripts are also added to the reference set with provisional nomenclature.

Separately the data from the YAN11293 sample were pre-processed using MiXCR v4.6.0, RepSeq.IO v2.4.0, MiLib v3.4.0 against ferret reference directory available at IMGT and filtered data were submitted on IMGT/HighV-QUEST.

For C genes, the same cDNA sequence data was analyzed on BLAST against IMGT reference directory and all hits with 100% identity and full-length coverage are shown in Table S2.

Results

Based on the analysis of the Iso-Seq cDNA data as described above, from the 96 ORF immunoglobulins (Ig) and T cell receptor (TCR) genes identified within ferret assemblies, we were able to change their functionality to F (functional) for 40 of them. For instance, IGHV7-5*01, which was described at the germline level with a non-canonical 'cacgatg' V-HEPTAMER was found with 100% identity at its full length, therefore it has been assigned as F within the IMGT database(https://imgt.org/IMGTgenedbdoc/dataupdates.html). As the same happens for nearly all IGHV7 subgroup genes (same heptamer), this heptamer has been declared canonical in the ferret. This heptamer is also found in *Neogale vison* (American mink) IGHV7 subgroup genes (https://www.imgt.org/IMGTrepertoire/LocusGenes/genetable/autotable.php?species=Neogale+vison&group=IGHV).

147 V genes, 14 D genes, 38 J genes were identified with 100% and full length compared to the germline in Ig and TCR cDNA sequences (Table S2). It should be noticed that some of the functional V, D, and J genes were not identified following our strict criteria (see 'V genes not found with cDNA support' in Table S2) potentially due to the somatic Ig and TCR recombination mechanisms which may involve trimming of nucleotides, or/and Ig somatic hypermutations, or because they were not expressed in the physiological conditions of our experiments the cDNAs were prepared and sequenced. Therefore, we believe based on the applied criteria that this number might be underestimated.

Concerning the C genes, IGHA*01 was found in several cDNA sequences; however it contains a substitution compared to the reference sequence in EX1. The secreted form of IGHE*01 was found with full-length and 100% identity to several cDNA sequences, however the membrane form is lacking, probably due to its low presence in cDNA. TRBC1*02, identified in the reference assembly, was not detected in cDNA sequences, however TRBC1*01, was previously identified in the assembly GCF_000215625.1. Consequently, we cannot exclude that TRBC1*02 might contain potential sequencing errors. All other constant genes from immunoglobulins and T cell receptors were found in full-length without mutations in cDNA (Table S2).

Additional supplemental tables

Table S1: Summary of the ferret Iso-Seq data and the abundance of ferret C-region isoforms (excel file).

Table S2: Ferret Ig and TCR C-region cDNA consensus sequences, annotations and description of allelic sites, and V, D, J gene annotations on the ferret reference genome assembly (excel file). The "IGHV Provisional" tab provides provisional names for IG V genes found on genomic contigs not linked with constant regions. The "Allelic Variants" tab describes allelic variants of IG and TR constant regions identified in transcriptomes and genomic sequences, including CCS counts for each allele. Transcriptomes from splenocytes from two (SRR29376976), four (SRR29376975), three (SRR29376974), or two (SRR26825671 and SRR26825672) were screened for the number of exact matches to the sequences as shown. Only those genes for which allelic variants were identified are shown here. Transcriptome datasets are available from BioSamples SAMN41805205, SAMN41805206, SAMN41805207, SAMN38182219 and SAMN38182220. Ferret Ig and TCR C-region annotations on the 10 additional ferret genome assemblies for the *in silico* PCR analysis of ferret-specific Ig and TCR primers are available on GitHub.

Table S3: Ferret Ig and TCR C-region specific primer sequences and the comparison of V(D)J contigs with primer hit and with vs. without the corresponding C-region match (excel file).

Table S4. Differentially expressed genes for each cell cluster of the ferret PBMC and splenocyte samples (excel file).

Table S5: Comparison of ferret cell type predictions using human references (in the same Supplementary Data file with Supplementary Figures).

Table S6: Functional enrichment analysis of differentially expressed genes between selected cell clusters identified in the ferret PBMC and Splenocyte samples (excel file).

Reference List

1. Cowell LG, Davila M, Kepler TB, Kelsoe G. 2002. Identification and utilization of arbitrary correlations in models of recombination signal sequences. Genome biology 3:research0072.1.
2. Merelli I, Guffanti A, Fabbri M, Cocito A, Furia L, Grazini U, Bonnal RJ, Milanesi L, McBlane F. 2010. RSSsite: a reference database and prediction tool for the identification of cryptic Recombination Signal Sequences in human and murine genomes. Nucleic acids research 38:W262-W267.
3. Gerritsen B, Pandit A, Zaaraoui-Boutahar F, Hout MCGNvd, Ijcken WFJv, Boer RJd, Andeweg AC. 2020. Characterization of the ferret TRB locus guided by V, D, J, and C gene expression analysis. Immunogenetics 72:101.
4. Wong J, Layton D, Wheatley AK, Kent SJ. 2019. Improving immunological insights into the ferret model of human viral infectious disease. Influenza Other Respir Viruses 13:535-546.
5. Alamyar E, Giudicelli V, Li S, Duroux P, Lefranc M-P. 2012. IMGT/HighV-QUEST: the IMGT® web portal for immunoglobulin (IG) or antibody and T cell receptor (TR) analysis from NGS high throughput and deep sequencing. Immunome Research 8:26.
6. Pegorier P, Bertignac M, Chentli I, Nguefack Ngoune V, Folch G, Jabado-Michaloud J, Hadi-Saljoqi S, Giudicelli V, Duroux P, Lefranc M-P, Kossida S. 2020. IMGT(R) Biocuration and Comparative Study of the T Cell Receptor Beta Locus of Veterinary Species Based on Homo sapiens TRB. Frontiers in immunology 11:821.
